# How Conditions and Resources Connected to Digital Management Systems and Remote Work Are Associated with Sustainable Work

**DOI:** 10.3390/ijerph192315731

**Published:** 2022-11-26

**Authors:** Andrea Eriksson, Lotta Dellve, Anna Williamsson, Katrin Skagert

**Affiliations:** 1 Division of Ergonomics, Department of Biomedical Engineering and Health Systems, KTH Royal Institute of Technology, 142 58 Stockholm, Sweden; 2 The Department of Sociology and Work Science, University of Gothenburg, 405 30 Gothenburg, Sweden; 3 Materials and Production Division, RISE Research Institutes of Sweden, 431 53 Mölndal, Sweden

**Keywords:** digital management systems, sustainable work, remote work, digital working conditions

## Abstract

The current state of work–life transformation will see more white-collar work being performed remotely using digital management systems. There is, however, a lack of research on factors and resources contributing to sustainable work when working remotely using digital management systems. The aim of this study was to study the conditions and resources connected to digital management systems and remote work, and their associations with sustainable work, in terms of process quality, trust, and sense of coherence, when working remotely during the COVID-19 pandemic. An analytical cross-sectional study was performed. Questionnaire data from white-collar employees (*n* = 484) in two private companies were analyzed with regression models, focusing on the importance of the conditions and resources connected to digital management systems and remote work, stratified by working from home or at the office. The results showed digital conditions and resources being associated with indicators of sustainable work. Furthermore, the results showed that social work relations were additional important explanatory factors for sustainable remote work. This study contributes to the development of a new post-pandemic work–life balance by concluding that sustainable remote work needs to be ensured by functional digital management systems and adequate leadership supporting the development of a positive team and learning climate.

## 1. Introduction

The COVID-19 pandemic contributed to many workplaces needing to radically reorganize work, meaning increased remote (mainly from home) work for white-collar workers. This has, in turn, meant an acceleration in work teams using cloud-based communication, documentation, and collaboration platforms, as well as an increased reliance on companies’ intranets and virtual private networks (VPNs), all hereafter referred to as digital management systems (DMSs). International studies during the pandemic point out that many employees would like to continue to work remotely at least some days a week [1,2]. Recently, many work organizations have also been implementing policies allowing employees to work remotely to a greater extent, compared to before the COVID-19 pandemic [3]. This implies that new technology in the form of DMSs will be critical for performing work. There is, however, limited research on critical resources and conditions that are important for DMSs contributing to sustainable work when working from home. The DMS functionality in terms of technical functionality and organizational support of employees’ functional work with DMSs will be used as an explanatory factor for outcomes related to sustainable work in this study.

Perspectives on sustainable work include the optimization and positive development of the work environment, quality of work, and efficiency of work [4]. This study focuses on possibilities to solve problems, improved efficiency, and improved production and processes as indicators of quality and efficiency in work. Sense of coherence (SOC) has been developed within salutogenic theory, focusing on factors and resources that promote people’s experiences of well-being [5]. Definitions of SOC at the workplace include that work is experienced as comprehensible (e.g., an understanding of situations and events that occur in work), manageable (e.g., experiences of having enough resources to perform work), and meaningful (e.g., motivational aspects including whether the work challenges are worth engaging in) [6]. Employees’ experiences of SOC and trust (including both trust between colleagues and trust between employees and management) will be used as indicators of a sustainable work environment.

Trust between employees and between managers and employees can be seen as a necessary condition for flexibility in when and where to work. The importance of trust for the success of virtual project teams working remotely has, for example, been highlighted [7]. A study on remote work during the COVID-19 pandemic reveals, however, that a substantial number of managers did not trust that employees would perform work efficiently when working remotely [8]. Trust can overall be defined as a critical work environment factor, as trust, both between employees and between managers, is associated with both well-being and work performance [9]. Overall, the idea of more trust-based governance of work has been raised as a reaction against the top-down control of work performance, which, for instance in health care, has been associated with increased stress at work and decreased quality of care [10,11,12].

The development of sustainable quality of work and work efficiency has also been highlighted as a process that promotes the development of individuals’ and organizations’ resources and resilience to dealing with different forms of work demands [4,13]. It has, in this context, been emphasized that the development of sustainable work requires a learning climate for developmental work, including a social team climate that promotes collaboration on work improvements [14]. This study thus includes, as explanatory factors, how digital resources, including DMSs, and a digital learning climate may contribute to or hinder outcomes related to sustainable work when working remotely.

An improved balance between work and private life, so-called work–life balance (WLB), is a strong contributing factor in wanting to continue to work from home even after the COVID-19 pandemic [1]. However, studies have shown that remote work for some may contribute to decreased WLB as working from home may erase boundaries between work and private life and may contribute to working beyond ordinary working hours [15]. Although earlier research has offered mixed results on whether there are any gender differences in experiences of WLB, some studies have shown that remote work specifically may contribute to less recovery time for women [15,16,17]. Overall, as WLB is an argument for increased flexibility in when and where to work, this study includes perceptions of flexible work arrangements supporting WLB as an explanatory factor for sustainable remote work. 

Several studies have pointed out that remote work is associated with improved individual work efficiency [18] and productivity [19,20], and greater autonomy in work [15,18]. However, reported disadvantages of working remotely include a lack of social contacts, unclear work conditions, and inadequate work tools [18]. A literature review has pointed out the specific challenges of maintaining relationships with colleagues and managers when working remotely [15]. In a qualitative study with employees working from home during the pandemic, some participants highlighted the lack of social contacts, including the lack of possibilities for creating a collective understanding of work, which in turn was experienced as contributing to work feeling less meaningful [21]. Managers themselves also reported challenges in supporting employees in gaining adequate achievements during remote work [8]. These results may imply that even if individual employees may have increased productivity and efficiency in performing specific work tasks remotely from work, deteriorated social relationships may hinder work goals that require collaboration and/or supervision. When working remotely, individuals’ own responsibility for leading and organizing work also increases. Relational resources as leadership and social capital in general are important resources for sustainable work [22]. Social capital in the workplace can, in this context, be defined as relations, networks, and reciprocity norms at the workplace that support collaboration on common goals [23,24]. Studies indicate that relational resources in the form of good contacts and cohesion with colleagues as well as supportive leadership practices contribute to both employees’ well-being and efficiency during remote work [15,17,25,26]. This study thus includes leadership and social capital as critical relational resources and explanatory factors for sustainable work when working remotely.

In summary, the current state of work–life transformation will see more white-collar work being performed remotely and using DMSs. There is, however, a lack of research on factors and resources that contribute to sustainable work when working remotely using DMSs. The aim of this study was to study conditions and resources connected to DMSs and remote work and how they are associated with sustainable work (in terms of process quality, trust, and SOC) when working remotely during the COVID-19 pandemic. The study aim was specifically to compare perceptions between those working mainly from home and those who to some extent worked at the office. The results from this study were drawn from questionnaire data collected from white-collar employees in two Swedish private companies. The result present perceptions of digital conditions, followed by the results of multilinear analysis on the importance of digital conditions and resources for work performance, trust, and SOC. The result contributes knowledge on critical resources and conditions for work organizations to consider when elaborating on flexibility for employees in when and where to work.

## 2. Materials and Methods

### 2.1. Study Design

An analytical quantitative cross-sectional study was performed. This meant that questionnaire data were collected at one point in time from white-collar employees in two private companies (companies A and B). It meant furthermore that data were analyzed with regression models, focusing on the importance of conditions and resources connected to DMSs and remote work, stratified by working from home or at the office.

### 2.2. Study Sample and Data Collection 

The study was part of a lager mixed method research project performed in Sweden with the overall aim to study critical leadership practices for sustainable work following the COVID-19 pandemic. Purposive sampling was applied. The aim was to include 300–500 employees from two companies representing different kinds of departments with white-collar workers who to different degrees had opportunities to perform work remotely from home. Two companies (companies A and B) were selected for the study based on the fact that they, following the COVID-19 pandemic, had implemented policies meaning flexibility for employees in when and where to work. 

Company A was a large global manufacturing company with approximately 13,500 employees in Sweden. Four departments with a total of 429 employees were selected for representing a variety of work areas, i.e., purchasing, IT, research and development, and safety and health. These departments had their white-collar office work and their geographic location in common but differed in type of work content and clients/customers. Company B was a Swedish real-estate company with about 400 employees spread geographically in different urban areas in Sweden. All employees at company B were included for participation as they represented white-collar workers with different work fields/educational backgrounds. Employees in company B worked mainly in white-collar types of work including administration, marketing, selling, rental, management, and attendance of properties. 

The research proposal was sent to the Swedish Ethical Review Authority for review. The authority decided no ethical review was necessary as no intervention on a research person was included in the study and as no personal information was treated in ways specified in the ethical review act. Informed consent was applied in the study. On the front page of the survey, the context and overall aim of the study were described, and it was stated that the individual consented to participate in the study by answering the survey.

In May 2021, a web-based questionnaire was sent out through email to the whole study population (*n* = 818), followed by four email reminders. The study sample consisted of total of 484 respondents from the two companies, which meant a response rate of 59%. Of these, 38% (*n* = 183) were women and 61% were men (*n* = 295). One person reported their gender identity as other. 

The survey in the overall research project consisted of approximately 80 survey questions including percentages of work time working remotely, aspects of and attitudes to flexible ways of working [27], usability and functionality of DMS modified after [28], digital learning climate inspired by [29], flexible work arrangements’ support of work–life balance [27] experiences of leadership, control and demands in work, trust and work engagement [30], social capital [31], SOC modified after [6], and process quality [32]. For this study, outcome variables representing sustainable work and explanatory variables representing conditions and resources connected to DMSs and remote work were selected; see specification of variables below. Furthermore, the survey question on percentage of working time performed remotely (in the pandemic context, meaning remotely from home) during the last month was selected. The response scale for this question was a visual analogue scale (VAS) ranging from 0 to 100.

### 2.3. Outcomes Variables

The outcome variables were as follows:

Trust (Cronbach’s alpha 0.84) consisted of an index including four items from COPSOQ III on perceptions of horizontal trust (between colleagues) and vertical trust (between employees and management [30]. The response scale was a five-point Likert scale ranging between “to a very large extent” and “to a very small extent”.

The unit’s improved process quality (Cronbach’s alpha 0.84) consisted of an index including three items concerning improvements in possibilities to solve problems, improved efficiency, and improved production and processes [32]. The response scale was a five-point Likert scale ranging between “to a great extent” and “there has been a deterioration”.

Sense of coherence (SOC) (Cronbach’s alpha 0.77) consisted of an index including three items on whether work is experienced as manageable, comprehensible, and meaningful [modified after 6]. The response scale was a seven-point Likert scale ranging between “to a very low degree” and “to a very high degree”.

### 2.4. Explanatory Variables—Digital Resources and Conditions

The following explanatory variables on digital resources and conditions were included:

Digital management system (DMS) functionality (Cronbach’s alpha 0.90) consisted of an index with nine items, including technical reliability, organizational support for employees’ work with a DMS, DMS functionality in supporting collaboration, communication, and performance of work [modified after 28]. The response scale was a five-point Likert scale ranging between “fully agree” and “fully disagree”. There was also an option to answer “do not know”. Digital learning climate (Cronbach’s alpha 0.92) consisted of an index with five items on the extent to which the work team discusses how DMSs impact work and how the teams’ collaboration on and work with DMSs can be improved inspired by [29]. The response scale was a five-point Likert scale ranging in between “fully agree” and “fully disagree”.

Flexible work arrangements’ support of work–life balance (WLB) (Cronbach’s alpha 0.77) consisted of an index including two items on whether flexible work arrangements (i.e., flexibility in when and where to work) supported individuals’ management of responsibilities and interests in and outside of work [27]. The response scale was a five-point Likert scale ranging in between “fully agree” and “fully disagree”.

### 2.5. Explanatory Variables—Relational Support Resources

The following explanatory variables on relational support resources were included:

Leadership quality (Cronbach’s alpha 0.89) consisted of an index from COPSOQ III with three items on perceptions of the closest manager’s ability to support development opportunities, planning work, and handling conflicts [30]. The response scale was a five-point Likert scale ranging between “to a very large extent” and “to a very small extent”.

Social capital (Cronbach’s alpha 0.91) consisted of an index with five items on collaboration and innovation climate as well as support and acceptance of each other within the work group [31]. The response scale was a five-point Likert scale ranging between “fully agree” and “fully disagree”.

### 2.6. Analysis

First, aspects related to the validity of the variables and data were analyzed. Cronbach’s alpha was calculated for each included index before proceeding with including the index in analysis. The resulting Cronbach’s alpha is written out for each index above. A variable was also created categorizing those who (a) worked remotely from home at least 90% or more of their working time (*n* = 235), and (b) worked at least 11% of the time at the office (*n* = 219). The sample size of the respondents working mostly remote was determined as a representative sample of the study population (i.e., the employees included in the study) based on a sample size formula for categorical data [33]. According to Ahmad and Halim [33], when having a study population of 800, a sample size of 200 persons is sufficient for categorical data (0.05 as margin of error and a t-value of 1.65) as well as for continuous data (at least 0.03 as margin of error and t-value of 2.58 or less). 

Descriptive analysis, bivariate correlations, and multilinear regressions analyses were performed. The statistical program JMP Pro 16 was used for analysis. A p-value less than 0.05 was set for determining statistical significance. Frequency tables with descriptive statistics were created to gain an overview of basic conditions, including time employed in current role, living situation, as well as extent of working from distance/at home before the pandemic. Bivariate correlations analyses were undertaken for all explanatory variables to check for multicollinearity. Correlations were categorized as follows: very strong (r = 0.81–1.00), strong (r = 0.61–0.80), moderate (r = 0.41–0.60), fair (r = 0.21–0.40), and weak (r ≤ 0.20) [34]. Data were plotted to assess normal distribution and linear relationships. Stepwise multilinear regressions analyses were performed stratified: for those working mainly remotely (e.g., mainly from home) and for those working more at the ordinary workplace. To further check for multicollinearity, the variance inflation factor was included in the multilinear regressions analyses. A cutoff value of five for the variance inflation factor was used to determine potential multicollinearity in the models, and all values for the variables in the models were below 2.0.

In model 1, variables representing aspects of DMSs and working remotely were included with the aim to analyze if the variables were statistically significantly associated with the outcomes and how much of the variance were explained by the variable. In model 2, relational aspects (leadership quality and social capital) were added with the aim to analyze if they had bigger or additional importance as explanatory factors for the outcomes. 

## 3. Results

### 3.1. Descriptives—Study Group and Digital Conditions

Table 1 gives an overview of the characteristics of the study group (*n* = 484). At the point of data collection, approximately half (52%) of the respondents worked remotely 90% or more of the time. Before the pandemic, about 5% of respondents worked remotely 90% or more of the time. Almost 10% lived in a single household, 14% lived with children aged 13–17, and around 25% lived with children younger than 13 years old. Almost half (47%) lived with another adult who also was at home during the daytime.

Thirteen percent had been employed one year or less, forty-three percent had been employed between 2 and 4 years, and forty-four percent had been employed five years or more.

Bivariate associations (see Table 2) showed moderate correlations between social capital and DMS functionality and weak correlation between flexible work support of WLB and leadership and social capital. Fair associations were shown for the other variables.

### 3.2. Importance of Digital Resources Conditions for Process Quality

The unit’s process quality was mainly explained by digital resources and conditions (model 1), both among those mostly working remote (*n* = 235) and more from office workers (*n* = 219). Relations added only 1–4% to the explained variance (r^2^, model 2). The importance of the explanatory variables for improved process quality differed depending on whether work was performed mostly remotely or more at the office. Improved process quality was to a greater extent explained by the variables’ flexible work support of WLB, DMS functionality, digital learning climate, and leadership quality (r^2^ = 0.32) for those working remotely. For the workers working more at the office, these variables were, to some extent, of lesser importance (r^2^ = 0.24), and the digital learning climate was not statistically significant. Detailed results are presented in Table 3. 

### 3.3. Importance of Digital Resources and Conditions for Trust and SOC

The digital resources and conditions in model 1 explained to a lesser degree the outcomes trust and SOC among workers working mostly remotely (r^2^ = 0.10–0.15). However, in model 2, to which relational factors were added, the explanatory variables explained the outcomes to a greater extent, especially for the outcome trust r^2^ = 0.41 for workers working mostly remotely (*n* = 235), and r^2^ = 0.35 for workers working more at the office (*n* = 219). Flexible work support of WLB was, however, not statistically significantly associated with trust. See Table 3 for detailed results. 

When the control variables were added to model 2, the results remained but indicated additional explanations related to experience. Years in work had positive importance for trust and SOC among those working more at the office but not for those mostly working remotely. 

## 4. Discussion

This study contributes knowledge on critical conditions and resources connected to DMSs and remote work, increasing the understanding of how they may have contributed to sustainable work when working remotely during the COVID-19 pandemic. Previous research has shown that remote work in a non-pandemic context is associated with improved individual work efficiency [18] and productivity [19,20]. The result of this study showed that digital conditions and resources in a pandemic context were associated with indicators of sustainable work, especially with process quality. The study further revealed that all explanatory factors, except social capital, were to a greater and significant extent associated with process quality in work for those working mostly remotely from home, as compared with those that still to some extent worked at the office. It is somewhat surprising that social capital was not significantly correlated to process quality as it includes assessments of the unit’s improved possibilities to solve problems, improved efficiency, and improved production and processes. Such improvements can be assumed to be dependent on key aspects of social capital, i.e., a collaborative and supportive climate for a common good. These results may, however, indicate that aspects such as DMS functionality and leadership quality were factors that were more critical for the possibility of keeping up work performance in the midst of a pandemic.

DMS functionality was furthermore associated with both process quality and SOC, especially for those working remotely. The results can be seen as expected as DMS functionality in this context includes aspects of technical reliability; organizational support for employees’ work with DMSs; and DMS functionality in supporting collaboration, communication, and performance of work [28]. It also underlines that aspects related to technical functionality of digital tools are important not only for productivity in work when working remotely but also with respect to employees’ experiences of SOC; e.g., that work is experienced as manageable, comprehensible, and meaningful. Previous research has shown that a lack of access to resources in the form of technical support, infrastructure, and tools hindered the development of satisfactory ways to perform remote work during the pandemic [21]. The results from this study imply the importance for work organizations to invest in the relation between DMS functionality and sustainable remote work.

The results revealed that flexible work support of WLB was associated with SOC for those working mostly remotely from home. Previous research has shown that improved WLB is a strong motivational factor for employees wanting to work remotely [1,18]. It is thus not surprising that flexible work supporting WLB was an explanatory factor associated with SOC. The results are in line with other research pointing at that flexible work life with respect to when and where one works may promote aspects of employees’ well-being [15]. However, remote work may mean increased individualization of work performance, including individual responsibility to create technical functional work environments. In this context, the result can be interpreted as leadership and social capital being additional relational resources linked to SOC. This suggests that flexible work arrangements are not enough for sustainable remote work but that work organizations need to invest in the development of relational resources when arranging flexible work opportunities. These relational resources may counteract the potential negative impacts for individuals working remotely [15,17,25,26], and the possible negative association with SOC for men working remotely may need further investigation in a longitudinal study.

Digital learning climate and DMS functionality were statistically associated with trust for both those working remotely and at the office. The results showed however that social resources in the form of leadership and social capital were more important for trust compared to the digital conditions and resources. This is in line with other research pointing to leadership and social cohesion in the work group as important antecedents of trust [35,36]. Previous research has highlighted that many managers distrust employees’ ability to perform work efficiently when working remotely [15]. One could argue whether true flexibility in when and where to work is not achievable without trust from managers and colleagues or if it is the opposite when it comes to performing work remotely. Trust may be seen as a key condition for work when initiating a project depending on digital team collaboration [7]. The results from this study indicate that work organizations offering opportunities for remote work may benefit from finding strategies for developing supportive teams and a leadership climate that nurtures trust. 

It is important to note that the pandemic forced rather than enabled home-based remote work. It was a radical change with no time to analyze, plan, and assess the fit of the change to either organizational contexts or individuals’ readiness for change, as is suggested for successful change [37]. In that way, the advantages of remote work in terms of having control over the organization of one’s own work may be lost. An overwhelming majority of participants in our study had not worked from home prior to the pandemic. Previous experience with remote work before the pandemic might have played a significant role in how to cope with the radically changed working conditions, as Donati and co-authors [37] showed in their cluster analysis. They showed that those who had experience of remote work before the pandemic and were working in big companies found remote work to be a more productive and effective way of working and exhibited positive attitudes related to remote work and their work–life balance [38]. 

This study was limited by its cross-sectional study design and was performed with employees in two selected companies, which may not have been a representative sample of white-collar workers in Sweden. There are, thus, limitations in terms of the generalizability of the results. However, the participants represented companies from two different kinds of businesses and organizational cultures, and they, furthermore, worked in different kinds of departments, representing different professional backgrounds and work assignments, thus mirroring a broad selection of different kinds of white-collar workers. The study took place in a Swedish context, including many employees having private, well-functioning broadband and many work organizations having started to implement DMS already before the COVID-19 pandemic. This means that DMS functionality might have been less of a hindering factor with respect to work compared to other national contexts. Additionally, the Swedish context stands out compared to other countries in how schools were kept open as much as possible and how home-schooling was only an exception. Research on white-collar workers’ remote work in Sweden during COVID-19, thus, can be reasoned to give an authentic example of probable remote work conditions without the COVID-19 pandemic should the study have been conducted elsewhere. 

## 5. Conclusions 

This study contributes with knowledge on what conditions and resources connected to DMSs and remote work and how they are associated with sustainable work (in terms of process quality, trust, and SOC) when working remotely during the COVID-19 pandemic. The study specifically analyzes differences in perceptions between those working mainly from home and those who to some extent worked at the office. The results reveal that the outcome process quality mainly was explained by digital resources and conditions. The results also point at that for the outcomes trust and SOC relational factors including leadership quality and social capital were additional important explanatory variables. This study contributes knowledge that digital conditions and resources are associated with indicators of sustainable work and underscore the importance of DMS functionality and digital learning climate when working remotely. The results, furthermore, indicate that social work relations are additional important explanatory factors for sustainable remote work. This means that leaders within work organizations need to ensure the functionality of DMS and ways to offer adequate leadership support and to develop a positive team climate if sustainable remote work is to be promoted. 

Future qualitative studies can be recommended to deepen the understanding of how relational resources, e.g., leadership support and team climate can be developed in companies, allowing for flexible work arrangements. Further qualitative studies are also recommended to study successful strategies for flexible work arrangements in ways supporting employees’ work–life balance.

## Figures and Tables

**Table 1:** Characteristics of the study group.

		All (*n*, %)	Remote Work >90% (%)	Office >11% (%)
All		484, 100%	52	48
Years in the work role	0–1 yrs	64, 13%	57	43
	2–4 yrs	208, 43%	57	43
	5–10 yrs	123, 25%	53	47
	11 + yrs	89, 18%	32	68
Living situation	Other adult at home	226, 47%	55	45
	Other adult not at home	197, 41%	49	51
	Children < 13 yrs	132, 27%	51	49
	Children > 13 yrs	69, 14%	69	31
	Living alone	46, 14%	52	48
Gender	Men	297, 61%	45	55
	Women	186, 38%	62	38
Company	Company A	245, 51%	44	56
	Company B	239, 49%	52	48

**Table 2:** Bivariate correlations.

		m/Md (SD)	1	2	3	4
Digital conditions	1. DMS functionality	3.68/3.78 (0.71)	1			
	2. Digital learning climate	3.23/3.2 (0.82)	0.34 ***	1		
	3. Flexible work support of WLB	3.65/3.5 (0.77)	0.21 ***	0.19 ***	1	
Relations	4. Leadership quality	3.62/3.67 (0.87)	0.37 ***	0.34 ***	0.12 *	1
	5. Social capital	3.85/4 (0.77)	0.39 ***	0.39 ***	0.15 **	0.46 ***

* = *p* < 0.05; ** = *p* < 0.01, *** = *p* < 0.001

**Table 3:** Linear models of the importance of digital tools and relational aspects for performance, trust, and SOC.

		Improved Process Quality		Trust		SOC	
		Remote Work > 90% (%) *n* = 181	Office > 11% (%) *n* = 152	Remote Work > 90% (%) *n* = 183	Office > 11% (%) *n* = 152	Remote Work > 90% (%) *n* = 160	Office > 11% (%) *n* = 136
		Beta	Beta	Beta	Beta	Beta	Beta
**Model 1**							
Digital conditions	DMS functionality	0.43 ***	0.19 *	0.17 *	0.24 ***	0.49 ***	0.27 *
	Digital learning climate	0.22 ***	0.17	0.22 ***	0.19 **	−0.03	0.19
	Flexible work support of WLB	0.22 ***	0.33 ***	−0.00	0.06	0.27 ***	0.16
	Intercept	0.19	0.98	2.78	2.27	3.05	3.50
	F-value	24.6	16.2	7.9	15.1	160	7.02
	R^2^	0.29	0.25	0.12	0.23	0.17	0.14
	R^2^Adj	0.28	0.23	0.10	0.22	0.15	0.12
**Model 2**		*n* = 178	*n* = 152	*n* = 180	*n* = 152	*n* = 158	*n* = 136
Digital conditions	DMS functionality	0.38 ***	0.16	−0.01	0.10	0.37 **	0.06
	Digital learning climate	0.15 *	0.17	0.04	0.05	−0.17	0.02
	Flexible work support of WLB	0.23 ***	0.35 ***	0.01	0.05	0.28 ***	0.17 *
Relations	Leadership quality	0.23 ***	0.15	0.20 ***	0.18 ***	0.26 ***	0.35 ***
	Social capital	0.00	−0.11	0.41 ***	0.26 ***	0.21 *	0.25 *
	Intercept	−0.21	0.92	1.65	1.66	2.17	2.54
	F-value	17.4	10.5	26.3	18.3	10.7	8.3
	R^2^	0.33	0.27	0.43	0.39	0.26	0.24
	R^2^Adj	0.32	0.24	0.41	0.36	0.23	0.21

* = *p* < 0.05; ** = *p* < 0.01, *** = *p* < 0.001. Controlled for gender, years in position, and living alone (0/1).

## Data Availability

The raw data supporting the conclusions of this article will be made available by the authors, without undue reservation.
